# Do bosons always condense?

**DOI:** 10.1093/nsr/nwaa219

**Published:** 2020-08-29

**Authors:** Yoshihiro Iwasa

**Affiliations:** Quantum Phase Electronics Center (QPEC) and Department of Applied Physics, The University of Tokyo, Japan; RIKEN Center for Emergent Matter Science (CEMS), Japan

We learn in elementary statistical mechanics that bosonic systems undergo a phase change even without interparticle interactions, called Bose-Einstein condensation (BEC). Indeed, almost all bosonic systems in the real world such as ^4^He, cold atoms and photons, exhibit BEC, and even electrons, a representative fermion system, can show similar condensation and superconductivity through the Cooper pair formation. Thus we have an impression that all bosonic systems undergo BEC, particularly in thermal equilibrium states. However, it seems there is no proof, and a natural question occasionally comes out: is this always the case, or is there any exceptional bosonic system that does not condense?

**Figure 1.**
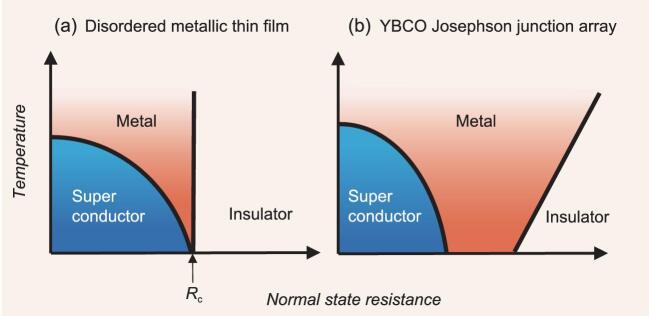
Schematic phase diagram of superconductors. (a) Disordered 2D superconductor [1] and (b) YBCO JJA system [2]. The horizontal axis, the normal state resistance, works as a controlling parameter. The conventional phase diagram (a) shows a quantum critical behavior at a critical resistance *R*_c_, while YBCO JJA (b) exhibits a broad metallic state at *T* = 0 K.

This question was first posed in superconducting systems at the end of the last century. Goldman *et al.* found that the two-dimensional (2D) superconductors (metallic thin films) exhibit a superconductor-insulator transition without an intervening metallic state [1]. Figure 1(a) shows a schematic phase diagram of their systems. This phase diagram is understood by a so-called dirty-boson model in terms of the quantum critical behavior. There, the superconductor-insulator quantum transition is controlled by the normal state resistance, a measure of disorder, because the film is highly disordered. Figure 1(a) convinced researchers that 2D bosons should either condense to a superconducting state or be localized. This conclusion was comfortable because it is consistent with the absence of noncondensing bosonic fluids.

In 2019, Jian Wang and his coworkers addressed this issue from a new viewpoint [2]. They created a thin film of high-temperature superconducting cuprate YBa_2_Cu_3_O_7-_*_x_* with well-aligned arrays of holes by means of reactive ion etching, so that the superconducting islands are resistively shunted. This can be regarded as a model system of Josephson-junction arrays (JJA). With increasing etching time, the link resistances grow and the superconducting islands shrink, which drives the system through a superconductor-anomalous metal-insulator transition. Their key achievement is the observation of magnetoconductance oscillation of all films including superconducting, metallic and insulating phases, with a constant period of *Φ*_0_ = *h*/2*e*. This provides firm evidence that the Cooper pair is taking a dominant role in these three states including insulators. In other words, the observed superconductor-metal-insulator transition is that of the bosonic system. They established a phase diagram on the plane of temperature and normal state resistance as shown in Fig. 1(b). The superconducting and insulating states are separated by the broad intervening metallic state. This shows a marked contrast with the conventional understanding of an absence of metallic state except for a single quantum critical point (Fig. 1(a)).

Recently, another approach has been taken using emerging 2D superconductors, where the crystallinity is dramatically improved owing to technological developments, including the exfoliation method, electric field effect and molecular beam epitaxy [3]. Such 2D superconductors were found to exhibit a ‘quantum’ metallic state once an out-of-plane magnetic field was applied, and this metallic state seemingly survived toward zero temperature without any condensation [4]. Similar behavior of JJA and 2D superconductors indicates the existence of bosonic systems which do not condense and stay in a normal fluid toward *T* = 0 K. These experimental observations will be a challenge to theories for further understanding of BEC.
